# Biomass 3D Printing: Principles, Materials, Post-Processing and Applications

**DOI:** 10.3390/polym15122692

**Published:** 2023-06-15

**Authors:** Yongxia Li, Xueyong Ren, Lin Zhu, Chunmiao Li

**Affiliations:** National Forestry and Grassland Engineering Technology Center for Wood Resources Recycling, College of Materials Science and Technology, Beijing Forestry University, Beijing 100083, China; lyx9801@bjfu.edu.cn (Y.L.); zhulin_1018@163.com (L.Z.); 13103189857@163.com (C.L.)

**Keywords:** biomass, 3D printing, material manufacturing, post-processing, applications, sustainable

## Abstract

Under the background of green and low-carbon era, efficiently utilization of renewable biomass materials is one of the important choices to promote ecologically sustainable development. Accordingly, 3D printing is an advanced manufacturing technology with low energy consumption, high efficiency, and easy customization. Biomass 3D printing technology has attracted more and more attentions recently in materials area. This paper mainly reviewed six common 3D printing technologies for biomass additive manufacturing, including Fused Filament Fabrication (FFF), Direct Ink Writing (DIW), Stereo Lithography Appearance (SLA), Selective Laser Sintering (SLS), Laminated Object Manufacturing (LOM) and Liquid Deposition Molding (LDM). A systematic summary and detailed discussion were conducted on the printing principles, common materials, technical progress, post-processing and related applications of typical biomass 3D printing technologies. Expanding the availability of biomass resources, enriching the printing technology and promoting its application was proposed to be the main developing directions of biomass 3D printing in the future. It is believed that the combination of abundant biomass feedstocks and advanced 3D printing technology will provide a green, low-carbon and efficient way for the sustainable development of materials manufacturing industry.

## 1. Introduction

Three-dimensional (3D) printing technology, also known as additive manufacturing, is a method that uses computer-aided manufacture to stack materials, layer by layer, to form a monolithic three-dimensional entity [1]. Additive manufacturing technology has the advantages of reducing the consumption of raw materials, saving production energy consumption, reducing time costs, etc. It has flexibility with regard to product production and is not limited by product shape and structure. Globally, 3D printing technology has been widely used in many fields such as manufacturing, construction, electronics, biomedical, aerospace, automotive [2,3], textile [4] and food [5].

According to the different material stacking principles, 3D printing technology can be divided into five categories [6]: (1) extrusion-based 3D printing technology, including Fused Filament Fabrication (FFF), Direct Ink Writing (DIW), Liquid Deposition Molding (LDM); (2) 3D printing technology based on UV beam scanning photosensitive resin, including Stereo Lithography Appearance (SLA), Digital Light Processing (DLP), Liquid-crystal display (LCD); (3) 3D printing technology based on powder sintering, including Selective Laser Sintering (SLS) and Selective Laser Melting (SLM); (4) 3D printing technology based on lamellar materials such as Laminated Object Manufacturing (LOM) and (5) 3D printing technology based on jet technology—3D inkjet printing is also called Three-Dimensional Printing (3DP). Different processes have different requirements for printing materials; 3D printing materials can be divided into four categories in terms of material types [7]: organic polymer materials, such as nylon, plastic and epoxy resin; metal materials, such as stainless steel, titanium, gold and silver; inorganic non-metallic materials, such as glass and ceramics; composite materials, such as cement and wood–plastic composites. Materials’ properties influence the printing process and final products. PLA is a crispy substance and has adverse performance and high stress relaxation in deformation recovery. Rahmatabadi et al. [8] added TPU to PLA to improve PLA’s 3D printing performance. The elastic properties of TPU made the composites have formability and a shape memory effect. 

**Figure 1:**
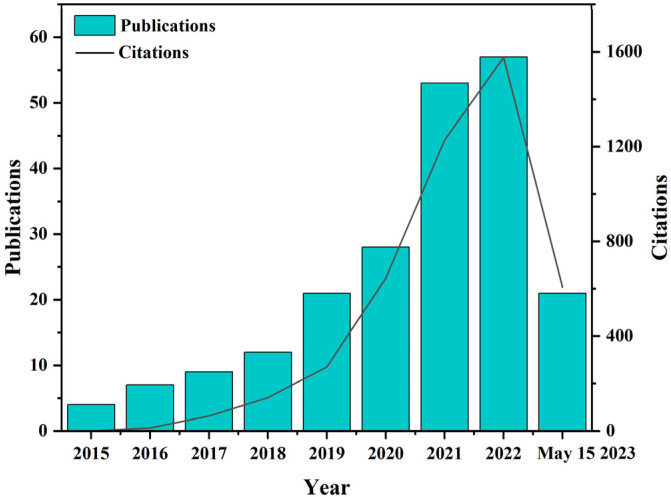
Number of publications and citations on Web of Science using the phrase “Biomass 3D Printing” from 2015 to 15 May 2023.

Biomass materials are mainly degradable materials made from woody, herbaceous, gramineous and vine plants, as well as their processing residues and waste, through physical, chemical and biological means. Biomass materials have the advantages of being green, widely sourced and cost-effective. They have broad development potential as green 3D printing consumables. In recent years, as the concepts of green and low-carbon have been emphasized, the research of biomass materials in 3D printing has been gradually developed and some comprehensive review articles have been published in this field. Figure 1 shows the research trends of biomass materials combined with 3D printing technology in recent years. The increasing number of publications and literature citations on 3D printing technology from 2015 to 15 May 2023 indicated that biomass 3D printing had become one of the hot issues. 

Previous review reports on biomass 3D printing have focused on the development of various types of 3D printing technology [9] and clarified the research progress of certain technologies [10,11,12]. The research on biomass materials in the field of 3D printing [13,14,15] as well as the application fields of biomass 3D printing have also been reported [16,17,18]. Tomec et al. [19] summarized a review of wood as one of the 3D printing consumable raw materials for FFF, inkjet printing, layered solid manufacturing and stereolithography and outlined research progress on 3D printed structures, architecture, continuous fiber printing, furniture design, four-dimensional printing and bionic products. Wood and lignocellulose material components can also be used as additives and reinforcements in composite materials [20]. Cellulose, hemicellulose, lignin and their derivatives have also been widely studied as the main bio-based raw materials for 3D printing technology [21]. Natural fibers have also shown unique advantages in FFF as well as in DIW [22,23]. In addition to agroforestry biomass materials, the advantages and limitations of algal biomaterials in 3D printing technology have been summarized and discussed [24]. 

However, there are few comprehensive articles on 3D printing technology, materials, post-processing and applications. In order to summarize the research progress of biomass 3D printing and provide a reference for a comprehensive understanding of 3D printing technology of biomass materials, this paper reviews the research progress of various biomass 3D printing technologies in recent years, the continuous enrichment of material types and the optimization process progress of post-processing. At the same time, the application fields of 3D printing biomass materials are also sorted out. Finally, the prospects of biomass 3D printing are identified.

## 2. Three-Dimensional Printing Technology Principles Using Common Biomass Materials

### 2.1. Principle of 3D Printing Technology

**Figure 2:**
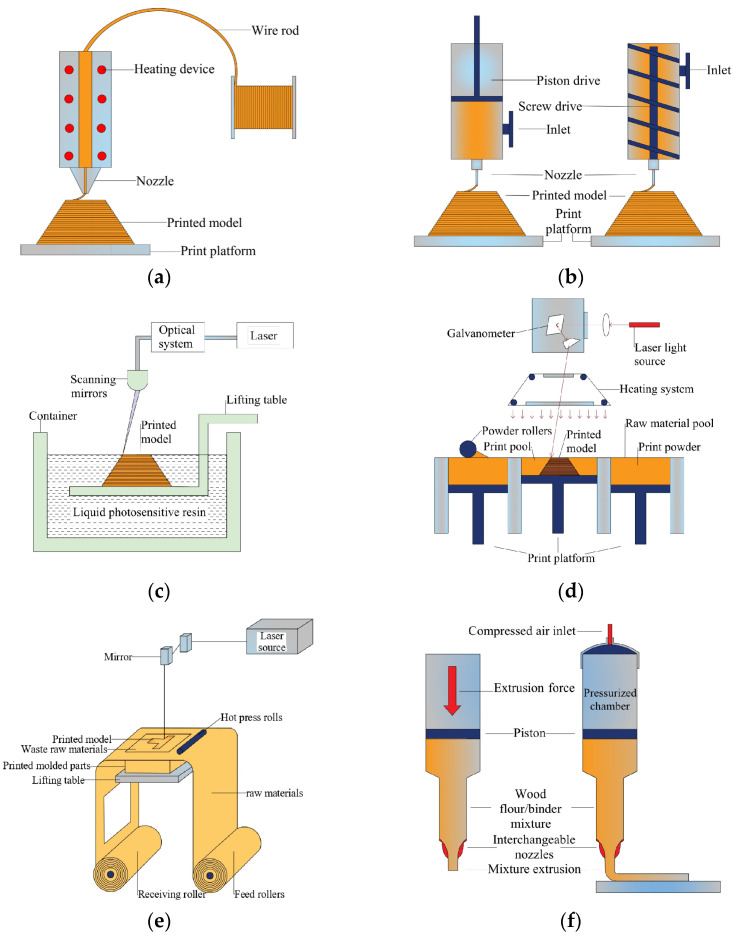
Six three-dimensional printing techniques’ schematic diagrams: (**a**) FFF, (**b**) DIW, (**c**) SLA, (**d**) SLS, (**e**) LOM, (**f**) LDM.

The data related to 3D printing technology come from three-dimensional model data based on computer design. After slicing the 3D model into 2D code information, it can be identified by the 3D printer. The principle of 3D printing technology is broadly defined as the accumulation of materials, layer by layer, to achieve free-form manufacturing, but different categories of 3D printing molding principles are distinguished. Figure 2 shows the principles of 3D printing technology commonly used for biomass materials.

FFF is an additive manufacturing technology based on the material extrusion process. The melted filamentous material is printed following a pre-designed moving path through a CNC precision nozzle to obtain the product. As shown in Figure 2a, the printed filament is cooled, bonded and cured to produce a two-dimensional plane and then the layers are stacked to complete the manufacture of three-dimensional objects.

As shown in Figure 2b, DIW is a computer-controlled process that relies on pneumatic pressure or screw extrusion. Under CNC operation, the high-viscosity paste is extruded to form a two-dimensional plane by a nozzle moving along a predetermined path and then a three-dimensional model is established by stacking layer by layer. 

The key to SLA is that the photosensitive resin is irradiated by UV light to complete the cross-linking and curing of the two-dimensional plane. Then, the printing platform is moved in the vertical direction and the cycle is repeated until it is printed as a three-dimensional entity. The printing schematic is shown in Figure 2c.

As shown in Figure 2d, SLS is a two-dimensional planar sintering by a predetermined selection zone when the melting point of wood powder is higher than the set temperature. The laser melts the plastic components to make them wrap the wood powder and bond them into a whole and then the layers are stacked through the movement in the Z-axis direction until they are formed.

LOM is a relatively mature additive manufacturing technology, first patented by Michael Feygin in 1985 and developed in 1986, after which development has continued for the past 37 years. As shown in Figure 2e, the feed component transmits the sheet coated with hot melt glue to the work table and the carbon dioxide laser beam is cut according to the data information. The supply compartment provides new materials, while the receiving compartment removes old materials. The material is bonded layer by layer until it is formed.

LDM is a new technology based on extrusion molding, as shown in Figure 2f. It uses a combination of wood chips or wood flour mixed with methyl cellulose and water or mixed with wood binders as raw material, which is deposited and molded under computer control.

### 2.2. Biomass Materials for 3D Printing

Notably, 3D printing technology requires materials to undergo liquefaction, powdering or filamentation under specific conditions. After printing, the interlayer adhesion and physicochemical properties must be suitable. Table 1 classifies biomass materials suitable for 3D printing technology in terms of three aspects: resource species, chemical composition and morphology.

## 3. Research Progress of 3D Printing Technology for Typical Biomass Materials

A variety of molding processes are included under the umbrella of 3D printing technology and this section summarizes the research progress of biomass materials in six common printing technologies. Table 2 lists the common materials used for biomass 3D printing technology with the advantages and disadvantages of each technology.

### 3.1. Fused Filament Fabrication (FFF) with Wood Plastic Filaments

Fused Filament Fabrication is a rapid prototyping technology. Wood plastic composites (WPCs) are a type of environmentally friendly bio-composite material made of thermoplastic compounded with wood or other biomass fiber materials (including wood flour, bamboo flour, straw, rice husk) and additives; they have been used as sustainable alternatives to natural timber products. WPCs have been studied extensively in the field of FFF and are among the main consumables of FFF technology.

**Figure 3:**
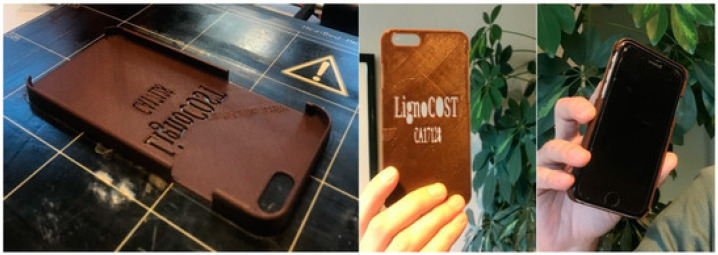
Illustrations of 3D printing of a smartphone protective case with PLA/lignin bio-composite filament [89].

Biomass materials, in the field of FFF, are usually mixed with other materials such as PLA and ABS to prepare composite materials. Xu et al. [88] prepared 3D printed biomass composites with shape memory effect by melt blending polyurethane, polycaprolactone and wood powder. Mihaela et al. [89] mixed lignin in spruce with melted PLA for 3D printing. The bio-composites had good extrusion and flowability when printing smartphone shells, as shown in Figure 3. This research showed the suitability of the biomass composite filament for 3D printing.

**Figure 4:**
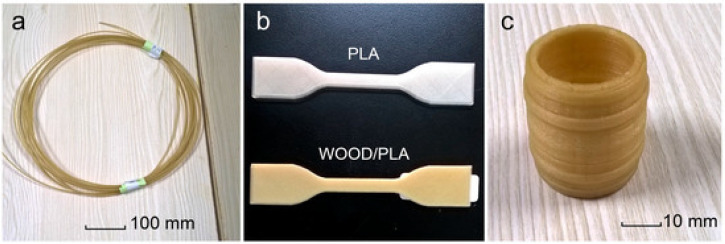
Filament, test specimens and 3D products: (**a**) WF/PLA composite filament; (**b**) specimens for tensile property measurement and (**c**) a barrel made by FFF 3D printer [92].

Materials used for FFF technology need to meet the basic requirements of flowability and adhesion in the molten state. Le Guen et al. [90] found that rice husk powder and wood flour have different effects on the rheological behavior as well as the overall stability of the compounds when mixed with PLA to prepare composite filaments. The addition of wood flour increased the composite viscosity of the compounds and the addition of rice husk powder decreased the complex viscosity of the compounds compared to the pure PLA material. To improve the interlayer adhesion of the printed materials, the lignin polymer was homogenized and modified with graphene nanosheets (GnPs) as reinforcing agents to prepare ABS particles, which were extruded in filament for FFF. The surface roughness and water contact angle of the printed samples were improved by 86% and 7.9%. The prepared composite material had a hydrophobic effect [91]. Tao et al. [92] mixed 5 wt.% of the wood powder with the PLA matrix to produce a compound filament, as shown in Figure 4. Compared with pure PLA, the composite with the addition of 5 wt.% wood powder had the texture of wood and good adhesion properties.

The factors affecting the printed products include the properties of the biomass itself, the percentage of biomass, the compatibility of composite materials and the processing parameters. Bhagia et al. [93] used poplar wood powder as a reinforcing material compounded with PLA for FFF to obtain excellent printing conditions by studying the size of the material, filament extrusion and printing method. It was found that the density of biomass was correlated with the composite density and the composite density affected the tensile strength of the material. The particle size of wood flour was also negatively correlated with tensile strength and the β-glycosidic bonding of polysaccharides in the biomass affected the Young’s modulus of the material. Bodaghi et al. [94] toughened PVC with biocompatible PCL. Composite materials have good compatibility, improved mechanical properties, excellent shape memory ability and 3D printability. Yu et al. [95] selected the residue of Astragalus membranaceus, a Chinese herbal medicine, to mix with PLA to prepare a wire for FFF and studied the influence of printing parameters on the performance of the object. The results showed that increasing the printing temperature and filling density or reducing the printing speed and layer thickness can improve the mechanical properties and thermal stability of the samples. Wang et al. [96] developed cellulose nanofiber (CNF) and PLA composite filaments for FFF. When the CNF addition was 2.5 wt.%, the resulting filament showed a smooth surface. When the CNF addition was greater or less than 2.5%, the mechanical properties of the molded parts decreased. The filament needed to be extruded from the nozzle after melting, which indicates that different materials require different preheating temperatures and the process needs to be adjusted according to the optimal temperature. If the material bonding force is poor, there will be a significant step effect, which will lead to a decline in mechanical properties. It can be concluded that layer classification is crucial in achieving the highest stiffness. Filling method and filling density have an effect on the surface quality and mechanical properties of 3D printed products [97].

### 3.2. Direct Ink Writing (DIW) with Biomass Gel Material

DIW is an additive manufacturing technology for rapid manufacturing of high-precision, multifunctional components. The key to researching this technology lies in the regulation of ink viscosity and fluidity to achieve the desired mechanical properties of the molded parts. The ink needs to have the ability of shear thinning and a certain energy storage modulus, allowing the material to be extruded smoothly from the nozzle and also maintain a stable shape after extrusion.

Markstedt et al. [71] prepared gels by dissolving bacterial nanocellulose (BNC) in the ionic liquid 1-ethyl-3-methylimidazole acetate. The gels exhibited shear thinning properties during printing and were self-supporting after extrusion. Thibaut et al. [98] used cellulose and carboxymethylcellulose (CMC) to prepare ink. The ink exhibited significant shear thinning behavior and yield stress after relaxation, making it suitable for 3D printing.

**Figure 5:**
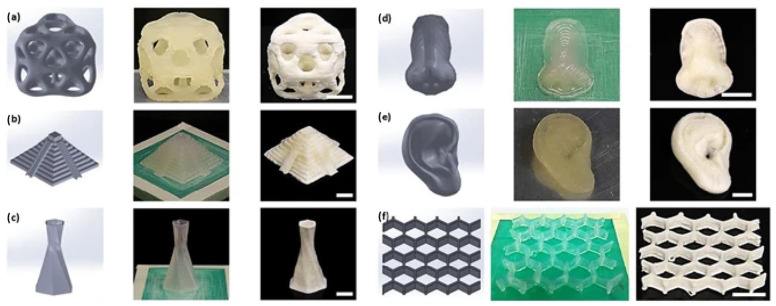
DIW 3D printed (**a**) octet cube, (**b**) pyramid, (**c**) hexagonally twisting vase, (**d**) nose model, (**e**) era model and (**f**) honeycomb from 20 wt.% CNC gel. The first column shows a CAD model, the second column shows DIW 3D printed gel structures and the third column contains the resultant structures after freeze drying. Scale bars are 1 cm [100].

In the biomass DIW printing process, biomass components account for a lower proportion. Post-treatment such as precipitation or freeze drying is required to remove the deposited liquid components to improve dimensional stability. Håkansson et al. [99] synthesized a hydrogel with shear thinning property composed of 2 wt.% cellulose nanofibers and 98 wt.% water for 3D printing. The resolution of the product was improved by a factor of 14 after removing the water by freeze drying. Vincent et al. [100] developed a pure cellulose nanocrystal aerogel with a unique inner pore structure, as shown in Figure 5. The increase in CNC content led to smoother printing edges, increased density and decreased porosity. After freeze drying, the CNC gel concentration was increased from 11.8 wt.% to 30 wt.%, with a porosity range of 75.0% to 92.1%.

Lai et al. [101] prepared nanocomposite gels with sufficient mechanical strength and tensile properties using cellulose nanocrystals (CNCs), deep eutectic solvents (DESs) and polyacrylic acid as raw materials. Then they used nanocomposite gels combined with an ionic cross-linking network to print sensors with good electrical conductivity. Hu et al. [102] fabricated a 3D printing ink using lignin. The rheological properties of the lignin-based ink varied from soft to hard, with self-supporting properties present when using triblock copolymers as cross-linking agents.

### 3.3. Stereo Lithography Appearance (SLA) with Biomass Resin Material

SLA is also known as stereolithography technology and the raw materials of SLA are mainly derived from petroleum substrates. In order to meet the shift in additive manufacturing toward a sustainable direction, exploring renewable raw materials has become an important research element.

**Figure 6:**
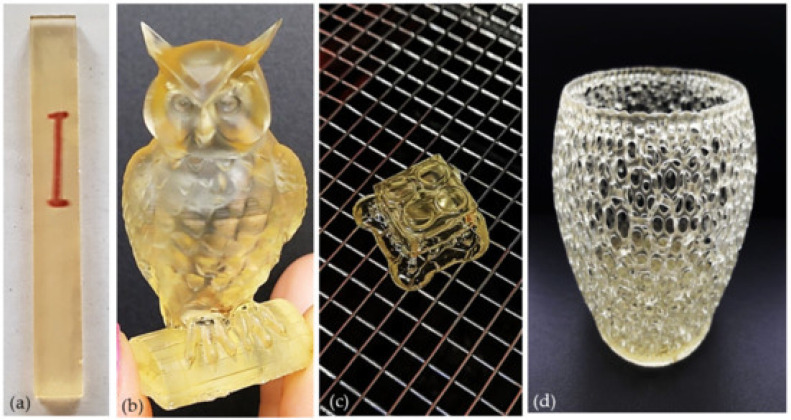
Printed M-AESO-3 resin: (**a**) transparent bar, (**b**) owl, (**c**) a Lego cube and (**d**) basket-like bowl [104].

Vegetable oils are renewable resources with high yield, biodegradability, low toxicity and surface modifiability which are highly promising and applicable in SLA. Anda et al. [103] prepared resin from acrylated epoxidized soybean oil (AESO) that could be used for SLA printing, which proves the great potential of vegetable oil in SLA. Figure 6 shows the printed products with the addition of reactive diluents.

Lebedevaite et al. [103] prepared photosensitive resins by blending AESO with vanillin dimethacrylate (VDM) produced from lignin. Both the homopolymer of AESO and the copolymer blended with VDM were suitable for SLA printing. In this study, rapid prototyping was realized by using Ultrashort pulses through multiphoton absorption and avalanche-induced cross-linking without using a photo-initiator, which promoted the development of 3D printing technology based on light. Tour’s team and Lin’s team [105] cooperatively synthesized inks with light-curing properties, biodegradability and renewability with soybean oil, natural polyphenols and luminescent graphene. They also succeeded in recycling the ink from printed products and upgrading the bio-composites into luminescent graphene. Romero et al. [106] reported the introduction of cork powder into a photocurable resin system to formulate a photosensitive resin. They investigated the effect of cork powder particle size on the mechanical as well as thermal properties of the composite, which improved with decreasing particle size.

Combining expensive and non-recyclable petroleum-based photosensitive resins with inexpensive and environmentally friendly biomass resins is an effective way to reduce material costs and promote sustainable renewability. Giuseppe et al. [107] blended petroleum-based resins containing urethane acrylates with 10–50 wt.% AESO for SLA technology. The resin had good thermal and swelling stability. This study demonstrated the feasibility of blending AESO with commonly used SLA resins. Guit et al. [108] developed a modified soybean oil light-curing resin and optimized the formulation to achieve 80% bio-based incorporation. The printed parts possessed great interlayer fusion and high print quality.

Sutton et al. [77,109] prepared photoactive acrylate resins by mixing commercially available light-curing resins with a 15 wt.% acylated lignin organic solvent. Subsequently, to improve the resin stability, the team modified the pine lignin by reduction and acylation to reduce its absorption of UV light. In the 3D printing range of 405 nm, the modified samples showed a 25–60% reduction in UV light absorption and an increase in stiffness and strength compared to the unmodified samples.

### 3.4. Selective Laser Sintering (SLS) with Wood Plastic Powder

The raw materials for SLS include nylon, polycaprolactone, sand, metal and wax, which are non-renewable resources. It is an urgent problem to find low-cost, green and biocompatible materials [110].

Guo et al. [111] successfully developed a rice husk plastic composite powder (RPC) using rice husk and PES. The obtained material met the SLS processing requirements in terms of mechanical properties and dimensional accuracy.

The content of wood powder particles is an important factor affecting the quality of SLS products. Li et al. [112] prepared materials suitable for SLS by mixing pine wood powder with phenolic resin. The content of pine powder in the mixture was 30–50 wt.%. The results showed that pine wood composites could be fabricated into highly customized carbon electrodes with high porosity by an SLS process.

In addition to the effect of biomass on the quality of molded parts, the process parameters of processing also have an important impact on the properties of the manufactured parts. Guo et al. [113] investigated the effect of laser intensity on the mechanical properties of wood–plastic composite molded parts. With the increase in laser intensity, the mechanical properties of the molded parts showed a trend of first increasing and then decreasing. When the laser intensity was less than 311 W/mm^2^, the PES interfacial adhesion and densification were improved with an increase in laser intensity. The impact strength of the parts also increased with the laser intensity.

The mechanical properties of biomass as a raw material for SLS are usually low and it is often necessary to add other materials or to incorporate post-treatment to improve their mechanical properties. Zhang et al. [114,115] aimed to solve the problem of low strength and low relative density of carbon nanotubes (CNT) and wood–plastic composites. Microwave treatment was used to post-treat the molded parts of SLS. The results showed that microwave treatment for about 60 s could improve the bending strength of CNT/WPC by 4.2–64.2%. Adding a small amount of Al powder as reinforcing material in WPC to accelerate the heat transfer could solve the problem of low mechanical properties of WPC.

### 3.5. Sheet-Based Laminated Object Manufacturing (LOM)

LOM is one of the rapid prototyping technologies. The biomass materials used mainly include paper sheets, WPC and wood veneer.

Sonmez and Hahn [116] analyzed the thermomechanical behavior of laminates during LOM printing and investigated the effects of process parameters on stress and temperature distribution in LOM technology. Small rollers caused concentrated stress distribution and larger diameter rollers were more conducive to bonding. The thickness of the laminate also affected stress distribution, with thinner laminates being more effective.

**Figure 7:**
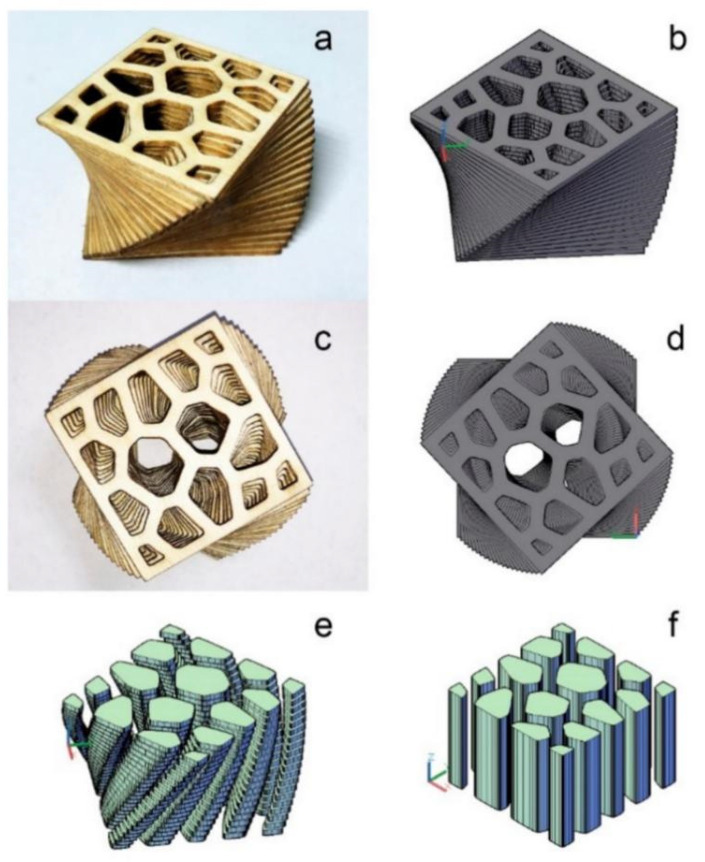
Comparison between the laser-cut veneer lamination (LcVL) product and its 3D model. (**a**) Orthographic view of the LcVL product; (**b**) Orthographic view of the 3D model of the product; (**c**) Top view of the LcVL product; (**d**) Top view of the 3D model of the product; (**e**) Tubular voids present in the 3D model of the product post-rotation; (**f**) Tubular voids present in the 3D model of the product pre-rotation [117].

Tao et al. [117] proposed a laser-cut veneer lamination (LcVL) technology based on LOM technology and plywood process, as shown in Figure 7, which glued cut wood veneers layer by layer and printed products with good surface quality and properties. This technology provided the basis for large-scale use of wood additive manufacturing.

### 3.6. Liquid Deposition Molding (LDM) with Biomass Paste Material

LDM is a hybrid extrusion-based molding process, which is a new method for 3D printing of biomass materials.

**Figure 8:**
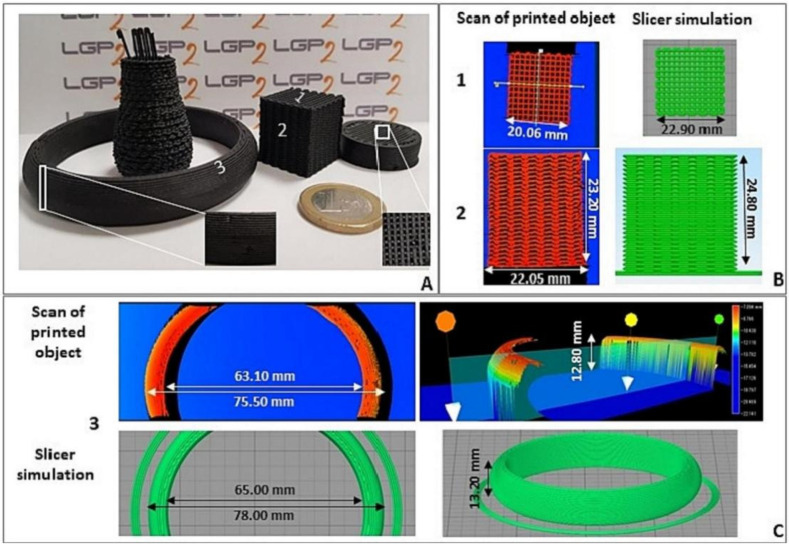
(**A**) A selection of printed objects, with a zoom-in on the possible defects, (**B**) real scans and simulation of a top view and lateral view of an unsupported printed cube and (**C**) a top view and 3D view of a filled printed ring. [63].

The properties of LDM-printed parts are largely determined by the type of binder. Kariz et al. [118] attempted to investigate the effect of beech wood powder and different types of binders on the quality of 3D printed molded parts. A mixture containing 17.5–20% polyvinyl acetate (PVAc) and a mixture containing 15–17.5% urea formaldehyde (UF) were selected for testing. Due to the high strength of UF adhesives, mixtures containing UF binders performed better than PVAc alone. Pitt et al. [119] investigated the application of wood waste in 3D printing. Glass fiber was used as reinforcement and urea–formaldehyde was used as a binder. The densification and in situ directional arrangement of the paste and fibers increased the tensile density of printed products by 73%. Bouzidi et al. [63] produced biomass-based composites for LDM using polyfurfuryl alcohol as a biomatrix and cellulose powder as a filler. The printed materials had good interlayer adhesion, mechanical properties and shape fidelity. Figure 8 shows the feasibility of printing complex structures. Rosenthal et al. [64] made a paste-like printing material using beech-milled wood chips and methyl cellulose dissolved in water. With methylcellulose as binder, the wood content could reach up to 90%.

The resolution, accuracy and surface finish of 3D printed objects are closely related to the selection of 3D printing technology. Different technologies have different printing principles, raw materials and post-processing processes. Different parameters for the same printing method can also lead to different results. Therefore, choosing the appropriate printing method is very important [120].

## 4. Post-Processing

Post-processing is an important procedure to further improve the surface quality as well as physical, chemical and mechanical properties of molded parts. Common post-processing methods for biomass 3D printed objects include drying treatment, curing treatment, heat treatment and surface modification treatment.

### 4.1. Drying Treatment

**Figure 9:**
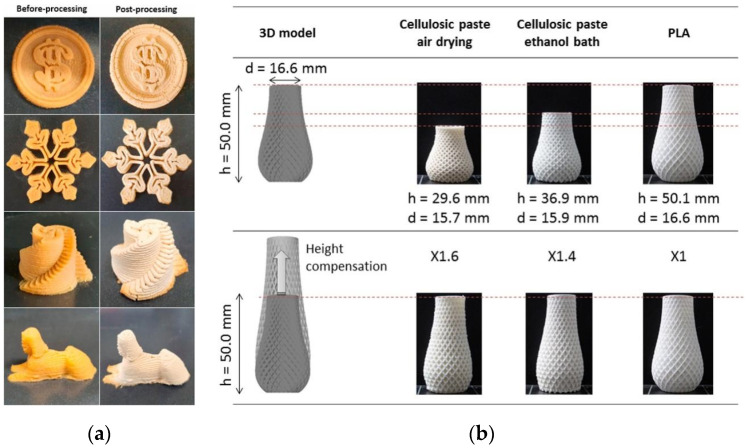
(**a**) Images of the 3D printed product after freeze drying post-processing [121]; (**b**) 3D printing of a double spiral vase with the optimized formulation dried with or without an ethanol bath or with polylactic acid (PLA). Upper row: 50 mm-high vase; Lower row: vase with height compensation in the model based on the calculated strain [98].

Drying treatment is mainly used for hydrogels and liquid slurries, making it a common post-treatment method for DIW- and LDM-printed products. Drying treatments include freeze drying, supercritical CO_2_ fluid drying, microwave drying and air drying. Different drying methods can lead to differences in the size and quality of the final product. Jo et al. [121] investigated the effect of freeze drying on the physical properties of 3D printed snacks. Figure 9a shows that freeze drying preserves the geometry of the article well, indicating that freeze drying-based post-treatment is a promising post-treatment technique for 3D printing. Tetik et al. [122] 3D printed using pure water as a supporting material. Water droplets froze and maintained their shape immediately after deposition. Then, by freeze drying to remove the supporting structure, an aerogel structure with good microstructure and pores was prepared. Thibaut et al. [98] developed a slurry for extrusion molding using natural cellulose fiber, carboxymethyl cellulose and distilled water as raw materials. The print vase was post-processed by air drying and the anisotropic deformation of air drying was eliminated by ethanol–water exchange before air drying. The printed vase is shown in Figure 9b.

### 4.2. Curing Treatment

Common curing methods include UV curing and thermal curing. UV curing is mainly used in photopolymer printing processes such as SLA, where the curing of photosensitive resins is achieved by UV light irradiation. Heat curing is mainly used for post-treatment of thermosetting resins. Garcia et al. [123] studied the effect of ultraviolet post-curing on the mechanical properties of materials. The results showed that the mechanical properties of materials were improved through curing treatment. Liu et al. [124] used AESO and modified ethyl cellulose monomer (ECM) as raw materials for 3D printing. They combined an extrusion process and UV post-curing. The printed product had good performance. Zhao et al. [125] synthesized a novel bio-based prepolymer–acrylate–epoxy tung oil polymer (AETP) for DLP 3D printing. Through UV post-curing, the product performance was improved. Thermal curing refers to the curing and cross-linking of thermosetting resins at a specific temperature. Chen et al. [126] printed ink containing UV-cured resin and epoxy oligomers through DIW. After the first stage of UV curing resin curing, thermal curing treatment was carried out. The mechanical properties of the cured material were excellent.

### 4.3. Heat Treatment

Heat treatment is mainly used in extrusion molding technology, commonly used in the treatment of liquid slurries and wires. The commonly used heat treatment methods for 3D printing molded parts include heating, annealing and partial melting. Untreated molded parts have an amorphous structure with a disordered arrangement of molecules. The extruded material cools rapidly and unevenly during the printing process, resulting in internal stresses. Annealing re-organizes the molecular structure of the uneven part, making it stronger and less prone to warpage. Partial melting is used to repair surface scratches caused by damage, support removal or surface post-treatment. The hot air is quickly passed through the area to be processed by a high-calorie heat gun to melt the surface and then recover an area similar to the original surface within a few seconds. Pascual-Gonzalez et al. [127] studied the effect of post-processing temperature on the performance of molded parts. Post-processing corrected dimensional deviations in the Z-axis of 3D printing technology. The increase in temperature enhanced the glass transition temperature of the specimen and reduced the plasticization of the specimen. Yang et al. [128] added CNC to methacrylate (MA) as a reinforcing material. The 3D printed composites were post-heated and the results showed that the post-treatment at 140 °C could promote the post-polymerization of nanocomposites and improve their mechanical properties.

### 4.4. Surface Modification Treatment

Surface modification treatment can generally be used for molded parts printed by various technologies and the method is selected according to actual needs. Common surface treatments include grinding, polishing, chemical impregnation, filling, painting, impregnating coating, powder coating and metal plating.

WPC and BPC are commonly used materials for FFF. The step effect is prone to occur in the printing process. Grinding and polishing are the most commonly used post-treatment methods for this technology and common materials [86]. Chemical impregnation refers to immersing the parts in chemical reagents and making the surface smooth through erosion. The pores generated during the FFF process can also be filled with epoxy resin or filler. The commonly used fillers in the market are paste fillers, spray fillers and resin fillers. Applying liquid paint is a common method, such as varnish, resin or plastic. Impregnation refers to immersing the object in paint, resin and paraffin so that the dip-coating material is uniformly coated on the surface of the print. A more recently developed post-treatment coating method also includes powder spraying. Powder spraying is also called rotary sintering. Each component is rotated and heated in the powder. If the powder encounters the heated part, the powder melts and adheres to the surface of the part to form a fine coating. Metal plating is a chemical method for bonding metal to the surface of 3D printed objects, which can obtain 3D printed products with high heat resistance, impact resistance, weather resistance and chemical resistance.

### 4.5. Other Post-Processing Methods

In addition to the above processing methods, the post-processing of biomass 3D printing objects can also be processed by pyrolysis, cold welding and ion solution processing. Pyrolysis is the decomposition of organic matter at high temperatures under low oxygen or anaerobic conditions. Cold welding is a solid-state welding process in which the welded interface does not need to be melted or heated. Cold welding does not produce a liquid or molten phase. Ionic solution treatment is a way of adding metal ions to the printing system and using ion interaction to induce ink gelation. Some researchers are actively exploring the post-processing process of 3D printed molded parts. Ge et al. [129] printed resorcinol formaldehyde resin by DIW and pyrolyzed the dried specimens to obtain the carbonization specimens. The results showed that the porosity and specific surface area of the sample after pyrolyzation was increased and the adsorption performance of the material was enhanced. For some special printing materials, taking ABS as an example, the problem of crushing can be solved by cold welding. If the ABS crushing part is treated with acetone, the surface is relatively flat [130]. Sun et al. [131] firstly 3D printed an acryloyl morpholine—acrylic acid–PEG diacrylate dry gel. Then, the dry gel was post-treated with ionic water solution to obtain water coagulation. The hydrogel obtained had a stable shape and good tensile properties.

## 5. Application

Of interest, 3D printing technology with biomass materials has a wide range of application areas. This section mainly summarizes the progress of 3D printed products in biomedical, electronics, construction, furniture, food and textile applications.

### 5.1. Biomedical

Biomass materials are widely used in biomedical fields due to their unique advantages of biocompatibility, biodegradability and low toxicity. The applications of biomass 3D printing in biomedicine mainly include printing artificial scaffolds [132], artificial tissues [133], wound healing [134] and drug carriers [135,136].

Miao et al. [137] studied the application of novel soybean oil epoxy acrylates in biomedical scaffolds. The material had shape memory capabilities and passed cytotoxicity tests. This material had better human bone marrow mesenchymal stem cell (hMSC) adhesion and value-added properties than conventional polyethylene glycol diacrylate (PEGDA). This study facilitated research on the combination of vegetable oils and other renewable chemicals to enable 4D printing.

**Figure 10:**
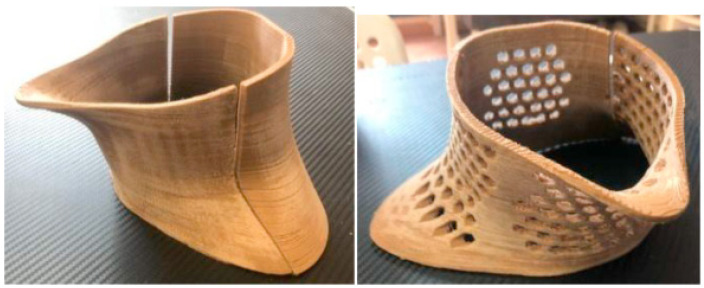
A 3D printed neck orthosis from hemp filament with a pattern of voids [138].

The combination of biomass materials and biomass-derived PLA provides non-toxic and environmentally friendly properties. Calì et al. [138] used PLA as the substrate and hemp with antibacterial properties as the reinforcement to manufacture silk. As shown in Figure 10, the neck orthosis was printed by 3D printing and the honeycomb shape had air permeability.

For some patients, taking multiple medications at once can cause discomfort. Biomass 3D printing can provide a solution to this problem. Khaled et al. [139] manufactured a multi-active solid dosage form from cellulose acetate and hydroxy propyl methyl cellulose. The hydrophobic cellulose acetate shell was extruded, which, thus, allowed the active drug to be mixed with hydrophilic hydroxy propyl methyl cellulose and filled into the segmented chambers of the cellulose acetate to form a sustained release chamber. Therefore, the combination of complex drug systems into customized tablets can be achieved, which will help patients who are currently taking multiple tablets.

Nanocellulose can make a transparent film to provide a humid environment for wound healing and can form elastic gels with bio-responsive properties. Rees et al. [140] printed wound dressings from carboxymethylated periodate oxidized nanocellulose. Since the material does not support bacterial growth, it provides conditions for its application in the medical field. Leppiniemi et al. [141] prepared nanocellulose alginate hydrogel wound dressings that could be used for 3D printing. Xu et al. [142] prepared biomimetic inks by mixing CNF with cross-linkable hemicellulose derivatives, which were used as a seeding matrix for human skin cells and pancreatic tumor cell cultures. The scaffolds showed good biocompatibility and shape fidelity. Avila et al. [143] printed auricular constructs containing human nasal cartilage (hNC) using nano-fibrillated cellulose and alginate (NFC-A) as raw materials. Jessop et al. [144] mixed CNF, CNC and alginate to prepare inks with rheological properties and biocompatibility that could be used for 3D printing of human nasal septal chondrocytes. The printed human nasal septal cartilage cells had high metabolism.

The combination of non-toxic and biosafety biomass materials with customized 3D printing provides an innovative approach for the medical field.

### 5.2. Electronics

Unlike traditional processing methods, 3D printing technology can print complex shapes. Biomass materials have been widely used in the field of electronics to produce, for example, supercapacitors [145], wearable jewelry [146] and sensors [147].

Nanocellulose has the characteristics of self-assembly and highly ordered orientation, which can improve its thermal conductivity. It has applications in supercapacitors, lithium-ion batteries and solar cell thermal management. The combination of CNF as a backbone material with carbon nanotubes, graphene and inorganic nitrides can be used to obtain composites with high thermal conductivity [148]. Cao et al. [149] 3D printed high-performance lithium metal batteries using CNF composites as raw materials. Yan et al. [150] prepared biomass-derived wood precursors with porous structure by SLS printing technique, which was combined with carbon thermal reduction techniques for structural–functional integration of electromagnetic wave absorption.

The substrates for flexible electronics are usually paper or polymer films. Hsieh et al. [151] prepared nano-paper for conductive circuits using CNF as raw material via 3D printing technology. CNF films have better porosity and smoothness than ordinary paper and their conductivity is enhanced to the level of lumpy silver.

**Figure 11:**
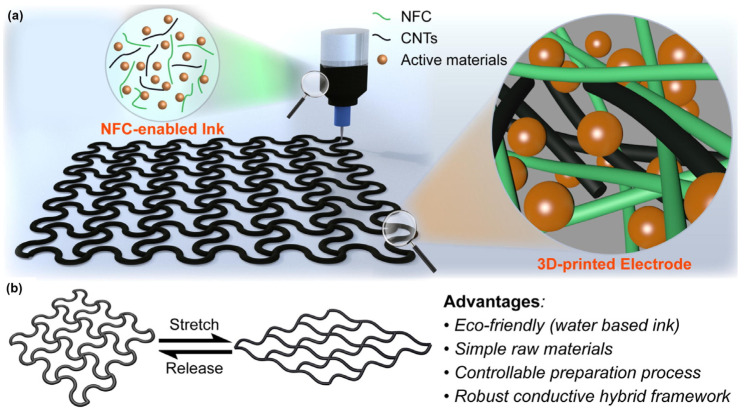
(**a**) Composition and morphological characteristics of 3D printing inks for the preparation of stretchable battery components, (**b**) The advantages of stretchable cells thus realized [153].

Sustainable development requires that the raw materials of electronic energy storage should gradually move towards renewability and environmental protection. Lignin (and its derivatives) and cellulose ether groups can prepare raw materials for 3D printing of electronic energy storage. Huang et al. [152] prepared hydrogels with great electrical conductivity and thermal sensitivity using hydroxyethyl cellulose (HEC) and polyvinyl alcohol (PVA) as backbone, borax as cross-linker and lignin as plasticizer. Li’s group at the University of Maryland, USA [153] prepared stretchable electrodes and diaphragms for lithium-ion batteries using nanocellulose as the active agent mixed with carbon nanotubes and active materials into aqueous inks. The printed serpentine structure gave the components excellent deformability. The high aspect ratio of cellulose and nanocellulose and the strong interaction between the components formed a robust nanoscale, demonstrating a huge application potential in the field of wearable and electronic energy storage devices. Figure 11 shows the basic situation of this stretched battery.

**Figure 12:**
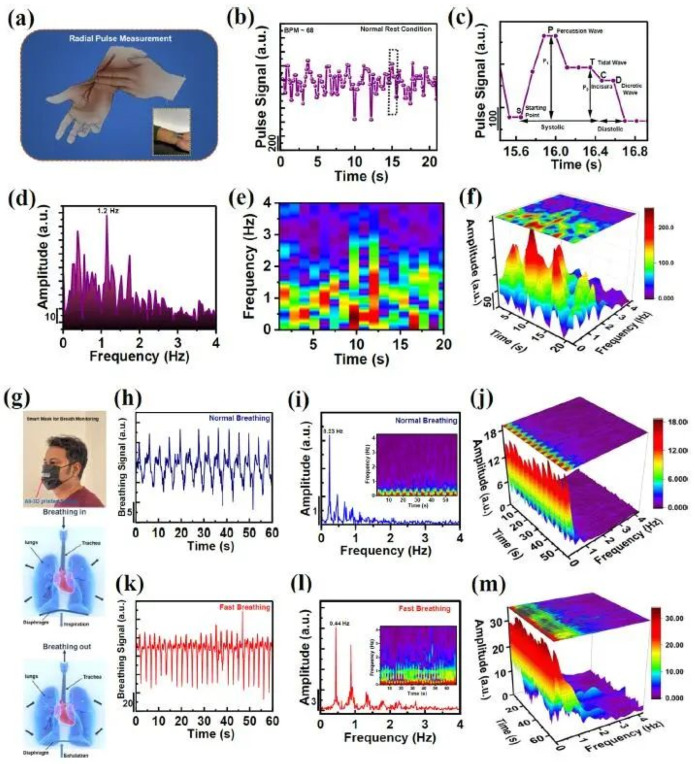
Self-powered radial pulse measurement and smart mask-based breath monitoring. (**a**) Schematic diagram of the typical radial pulse measurement. (**b**) Corresponding radial pulse signal measured at the normal rest condition. (**c**) Typical radial pulse spectra of the one cycle. The FFT-processed frequency spectrum: amplitude over frequency in (**d**) and frequency over time in (**e**). (**f**) The STFT-processed 3D spectrogram. (**g**) Photo showing the all-3D printed sensor attached for smart mask-based breathing. Respective output electrical signal, recorded under (**h**) normal breathing and (**k**) fast breathing. The FFT-processed frequency spectrum: (**i**) normal breathing and (**l**) fast breathing. The STFT-processed 3D spectrogram: (**j**) normal breathing and (**m**) fast breathing [154].

Saha et al. [154] fabricated a Pyro-Piezoelectric Nanogenerator (Py-PNG) by 3D printing CNC. The Py-PNG had good thermal energy collection characteristics and sensitivity to accurately monitor the heart and lungs and also paved the way for the application of temperature sensors and power generation. Figure 12 shows how the program was applied and how the lungs behaved during breathing.

### 5.3. Construction Field

**Figure 13:**
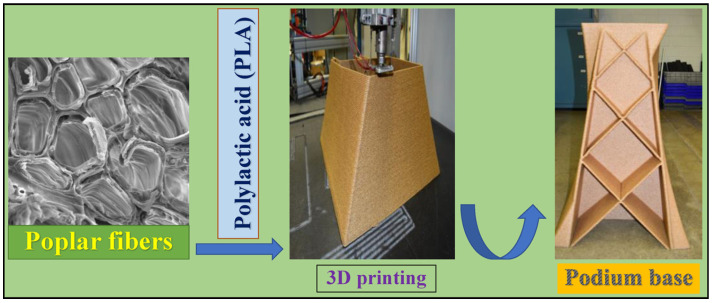
A large 3D printer was used to successfully create a podium base from poplar/PLA composites [157].

For construction, 3D printing technology has the characteristics of personalization, high material utilization and independent molding, which gives it advantages in the field of large-scale component printing. Thus, 3D printing technology can provide models for early design in the construction field and provide designers with the opportunity to design flexibly. The building base and building materials can also be achieved through 3D printing. Klaudius et al. [155] used a mixture of wood-based bulk materials (sawdust, wood chips, etc.) and adhesives (gypsum, cellulose, sodium silicate and cement) for 3D printing. Finally, materials with comparable performance to kapok lightweight construction panels were obtained. Yoshida et al. [156] proposed a method of 3D printing a building model and successfully printed the venue model. Building materials were composed of ulna and glue made of Chinese fir. Zhao et al. [157] successfully printed the base of a building using poplar fiber and PLA as the raw materials for 3D printing. The properties were stable and controllable, as shown in Figure 13. The emergence of concrete in the construction field has led to a reduction in the use of soil-based materials and an increase in greenhouse gas emissions. In search of greener building materials, Alqenaee et al. [158] used a mixture of straw, water, sand and clay for 3D printing. By changing the material ratio, the printed mixture had better tensile and compressive properties, which indicates that the modified material has the potential to print houses.

### 5.4. Furniture Field

**Figure 14:**
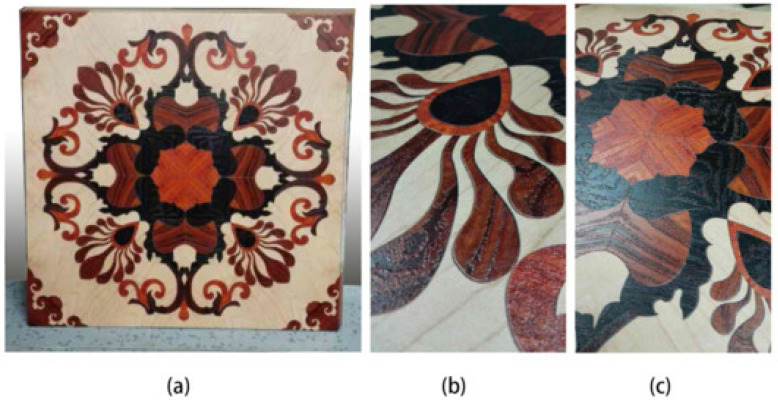
Printing marquetry designing on MDF: (**a**) square digital printing marquetry board with installation notch, (**b**) details of printing 3D wood texture and (**c**) replicated wood marquetry [162].

The application scope of 3D printing technology in the furniture manufacturing industry is expanding, from single-piece furniture production to mold manufacturing, product development and parts’ manufacturing [159]. Traditional furniture mainly relies on mold manufacturing. The personalized and complex shape manufacturing characteristics of 3D printing enable furniture to present various shapes and make furniture components easy to assemble and disassemble, which is very suitable for the field of intelligent furniture. As well, 3D printed wood products have a natural texture, strength, hardness, precision and finish that are suitable for furniture and office furnishings. For example, 3D printed WPC products not only have wood texture, but also have a certain scratch-resistant line and wear resistance [160]. As shown in Figure 14, wood textures can be printed directly on the panels through 3D printing technology to imitate the effect of handicraft works and enhance aesthetics [161,162].

### 5.5. Other Fields

Some 3D printed cellulose structures have good mechanical properties and excellent flexibility for textile applications. Wang et al. [96] developed composite filaments with high tensile strength and elongation at break using CNF and PLA as raw materials. The hydrophilicity of CNF is conductive to later coloration, laying the foundation for its application in textile and apparel fields.

Notably, 3D printing technology can meet the requirements of efficient and personalized food processing. CNF is also widely studied as a dietary fiber for developing healthy, customizable snack products. The use of starch with other materials for 3D printing has become very common.

Martina et al. [163] extracted CNF from bleached sulfate birch pulp, mixed it with starch, skim milk powder and semi-skimmed milk powder and, finally, manufactured food via 3D printing. Shahbazi et al. [164] introduced multifunctional microcrystalline cellulose with good antioxidant activity into soy protein to obtain printing ink with pseudoplasticity and viscoelasticity. This study provided ideas for developing low-fat 3D printed artificial meat. Letras et al. [165] created a gluten-free cereal snack from Chlorella and Spirulina through 3D printing techniques. Printed snacks had high protein and essential mineral content, according to nutritional characteristics. Nida et al. [34] printed food packaging shells from sugarcane bagasse which could be used as an alternative to petroleum-based materials.

In short, the development of 3D printing technology has led to simultaneous progress in various fields of social life.

## 6. Prospects

As demonstrated, 3D printing technology has enormous development potential and will open up new ways for the utilization of biomass resources. The future development of biomass 3D printing needs to focus on the following aspects.

Firstly, the types of biomass materials for 3D printing need to be further expanded. Although biomass has been studied and applied in 3D printing technology, the proportion of biomass in 3D printing raw materials is relatively small. This makes it difficult to fully utilize the advantages of low price, sustainability and widespread availability of biomass resources. In future research, it is necessary to increase the proportion of biomass materials in 3D printing raw materials, or to develop more materials entirely derived from biomass. At the same time, improving the mechanical properties, stability, heat resistance, durability and compatibility of biomass materials with other materials is imminent. The addition of functional properties to biomass 3D printing materials should also be considered.

Secondly, it is necessary to further enrich the types of 3D printing technology and update the existing technology to be suitable for biomass 3D printing. At present, the main 3D printing methods applicable to biomass are the six technologies mentioned above and it is crucial to continue developing 3D printing technologies suitable for biomass materials. In order to further develop biomass 3D printing, consideration should also be given to reducing 3D printing costs and simplifying operating procedures. Improving the printing speed and accuracy of 3D printing technology to meet higher end, finer and more complex applications is also an area that needs to be explored.

Finally, it should be put on the agenda to speed up the application field of biomass 3D printing products. The non-toxic and environmentally friendly characteristics of biomass resources can provide convenience for their use in various fields. With the development of biomass chemistry and processing technology, 3D printing technology using biomass as a raw material will be able to customize and mass-produce according to different needs.

## 7. Summary

The rapid development of 3D printing technology is a major change in the manufacturing industry worldwide. In the context of the era of “carbon peak” and “carbon neutrality”, the combination of biomass materials and 3D printing technology provides an innovative, practical and sustainable direction. Biomass resources have been applied in various technologies of 3D printing and the types of biomass materials used in 3D printing are also constantly enriching. The post-treatment of 3D printed specimens of biomass also includes various methods such as drying, solidification, heat treatment and surface modification, which can improve the performance of printed specimens. It is not an exaggeration to say that the advantages of 3D printing technology, such as high material utilization, low energy consumption and personalized customization, combined with the sustainable, non-toxic and low-cost advantages of biomass, will have a significant impact on the sustainable development of society and promote continuous progress in fields such as biomedicine, electronic equipment, food, textiles, architecture and furniture. The research on biomass 3D printing has just begun and will achieve significant breakthroughs in the near future.

## Figures and Tables

**Table 1:** Summary of biomass materials being used for 3D printing.

Classification Basis	Category	Biomass Materials		Printing Method
Resource species	Marine Biomass	Sodium alginate [25], Agarose [26]		DIW
	Forestry Biomass	Coniferous wood [27], Broadleaf wood [28], Bamboo wood [29]		FFF, DIW, SLS, SLA, LOM, LDM
	Agricultural biomass	Straw [30], Wheat [31], Rice [32], Corn [33], Sugar cane [34]		FFF, DIW, SLS, SLA, LOM, LDM
Chemical component	Mono-component biomass materials	Cellulose	Nanocellulose [35], Cellulose nanofiber [36], Cellulose nanocrystals [37], Cellulose acetate [38], Nano-fibrillated cellulose [39], Microcrystalline cellulose [40]	FFF, DIW, SLS, SLA, LOM, LDM
		Hemicellulose	Hemicellulose paste [41] Galactoglucomannan [42], Hemicellulose hydrogel [43]	DIW, FFF
		Lignin	Lignin powder	FFF [44], SLS [45]
			Sulfonate lignin [46]	FFF
			Lignin hydrogel [47]	DIW
		Other	Chitosan [48], Soy protein [49], Starch [50]	DIW
	Composite biomass materials	Compound of wood with plastic [51] Bamboo compounded with plastic [52] Compound of straw with plastic [53] Compound of cellulose [54]/lignin [55] with plastic		FFF, SLS
Form and morphology	Wires	Wood plastic wire [56]		FFF
	Particles	Wood plastic pellets [57]		SLS
	Gel	Nanocellulose hydrogel [58] Cellulose ionic liquid [59]		DIW
	Resin	Epoxy acrylate soybean oil (AESO) [60],		SLA
	Sheet	Paper [61], Wood veneer [62]		LOM
	Chips and powder	cellulose powder [63], Wood chips [64]		LDM

**Table 2:** A list of 3D printing technologies for biomass materials and their respective applicable raw materials, advantages and disadvantages.

Technology	Applicable Biomass Materials	Advantages	Disadvantages
FFF	Bamboo plastic composite [52] Wood plastic composite [65]	Easy operation of the equipment [66], Simple post-processing [67], Wide range of molding materials [68]	Low accuracy of molded parts [69], Support structure required, Slow molding speed, Nozzle easily clogged [70]
DIW	Hemicellulose hydrogel [43] Lignin hydrogel [47] Cellulose ionic liquid [71] Cellulose hydrogel [72] Cellulose derivative hydrogels [73]	No support required [74], Wide range of raw material sources [75], Higher accuracy [76]	Complex post-processing [76]
SLA	Vegetable oil-based photosensitive resin [60] Lignin-based photosensitive resins [77]	Fast molding speed, Highest printing accuracy [78]	Poor environmental protection of raw materials [10] Restricted material types [79],
SLS	Wood Plastic Composite [57]	Simple process, No support structure required [80], High raw material utilization [81]	Complex post-processing [82], Rough and porous forming surface, Requires preheating and cooling [83]
LOM	Paper [61] Wood veneer [62]	Low price of raw materials [84], Fast molding speed, High printing accuracy, No support structure required [85]	Complex post-treatment process [86]
LDM	Cellulose powder [63] Wood chips [64],	Easy operation of the equipment, Lower cost [87]	Restricted material types [63] Post-processing required [86]

## Data Availability

Not applicable.

## References

[1] Ngo T.D., Kashani A., Imbalzano G., Nguyen K.T.Q., Hui D. (2018). Additive manufacturing (3D printing): A review of materials, methods, applications and challenges. Compos. Part B Eng..

[2] Tumer E.H., Erbil H.Y. (2021). Extrusion-Based 3D Printing Applications of PLA Composites: A review. Coatings.

[3] Dasgupta A., Dutta P. (2022). A Comprehensive Review on 3D Printing Technology: Current Applications and Challenges. Jordan J. Mech. Ind. Eng..

[4] Dip T.M., Emu A.S., Nafiz M.N.H., Kundu P., Rakhi H.R., Sayam A., Akhtarujjman M., Shoaib M., Ahmed M.S., Ushno S.T. (2020). 3D printing technology for textiles and fashion. Text. Prog..

[5] Rong L.Y., Chen X.X., Shen M.Y., Yang J., Qi X., Li Y.L., Xie J.H. (2023). The application of 3D printing technology on starch-based product: A review. Trends Food Sci. Technol..

[6] Mazurchevici A.D., Nedelcu D., Popa R. (2020). Additive manufacturing of composite materials by FDM technology: A review. Indian J. Eng. Mat. Sci..

[7] Jimenez M., Romero L., Dominguez I.A., Espinosa M.D., Dominguez M. (2019). Additive Manufacturing Technologies: An Overview about 3D Printing Methods and Future Prospects. Complexity.

[8] Rahmatabadi D., Soltanmohammadi K., Pahlavani M., Aberoumand M., Soleyman E., Ghasemi I., Baniassadi M., Abrinia K., Bodaghi M., Baghani M. (2023). Shape memory performance assessment of FDM 3D printed PLA-TPU composites by Box-Behnken response surface methodology. Int. J. Adv. Manuf. Technol..

[9] Alghamdi S.S., John S., Choudhury N.R., Dutta N.K. (2021). Additive Manufacturing of Polymer Materials: Progress, Promise and Challenges. Polymers.

[10] Maines E.M., Porwal M.K., Ellison C.J., Reineke T.M. (2021). Sustainable advances in SLA/DLP 3D printing materials and processes. Green Chem..

[11] Bardot M., Schulz M.D. (2020). Biodegradable Poly (Lactic Acid) Nanocomposites for Fused Deposition Modeling 3D Printing. Nanomaterials.

[12] Azad M.A., Olawuni D., Kimbell G., Badruddoza A.M., Hossain M.S., Sultana T. (2020). Polymers for Extrusion-Based 3D Printing of Pharmaceuticals: A Holistic Materials-Process Perspective. Pharmaceutics.

[13] Dai L., Cheng T., Duan C., Zhao W., Zhang W.P., Zou X.J., Aspler J., Ni Y.H. (2019). 3D printing using plant-derived cellulose and its derivatives: A review. Carbohydr. Polym..

[14] Wang Q.Q., Sun J.Z., Yao Q., Ji C.C., Liu J., Zhu Q.Q. (2018). 3D printing with cellulose materials. Cellulose.

[15] Gauss C., Pickering K.L., Muthe L.P. (2021). The use of cellulose in bio-derived formulations for 3D/4D printing: A review. Composites Part C Open Access.

[16] Lin L., Jiang S.L., Yang J., Qiu J.D., Jiao X.Y., Yue X.S., Ke X.R., Yang G.J., Zhang L. (2023). Application of 3D-bioprinted nanocellulose and cellulose derivative-based bio-inks in bone and cartilage tissue engineering. Int. J. Bioprint..

[17] Chinga-Carrasco G. (2018). Potential and Limitations of Nanocelluloses as Components in Biocomposite Inks for Three- Dimensional Bioprinting and for Biomedical Devices. Biomacromolecules.

[18] Li G.S., Hu L.P., Liu J.L., Huang J.Y., Yuan C.H., Takaki K., Hu Y.Q. (2022). A review on 3D printable food materials: Types and development trends. Int. J. Food Sci. Technol..

[19] Tomec D.K., Kariz M. (2022). Use of Wood in Additive Manufacturing: Review and Future Prospects. Polymers.

[20] Lamm M.E., Wang L., Kishore V., Tekinalp H., Kunc V., Wang J.W., Gardner D.J., Ozcan S. (2020). Material Extrusion Additive Manufacturing of Wood and Lignocellulosic Filled Composites. Polymers.

[21] Yang J., An X.Y., Liu L.Q., Tang S.Y., Cao H.B., Xu Q.L., Liu H.B. (2020). Cellulose, hemicellulose, lignin, and their derivatives as multi-components of bio-based feedstocks for 3D printing. Carbohydr. Polym..

[22] Bi X., Huang R. (2022). 3D printing of natural fiber and composites: A state-of-the-art review. Mater. Des..

[23] Ahmed W., Alnajjar F., Zaneldin E., Al-Marzouqi A.H., Gochoo M., Khalid S. (2020). Implementing FDM 3D Printing Strategies Using Natural Fibers to Produce Biomass Composite. Materials.

[24] Mandal S., Nagi G.K., Corcoran A.A., Agrawal R., Dubey M., Hunt R.W. (2023). Algal polysaccharides for 3D printing: A review. Carbohydr. Polym..

[25] Abouzeid R.E., Khiari R., Salama A., Diab M., Beneventi D., Dufresne A. (2020). In situ mineralization of nano-hydroxyapatite on bifunctional cellulose nanofiber/polyvinyl alcohol/sodium alginate hydrogel using 3D printing. Int. J. Biol. Macromol..

[26] Fan R., Piou M., Darling E., Cormier D., Sun J., Wan J.D. (2016). Bio-printing cell-laden Matrigel-agarose constructs. J. Biomater. Appl..

[27] Tsai M.T., Wang P.C. (2023). Application of lignocellulosic composite (Taiwan incense-cedar) for digital light processing (DLP) in 3D printing. Wood Mater. Sci. Eng..

[28] Zhang S.Y., Bhagia S., Li M., Meng X.Z., Ragauskas A.J. (2021). Wood-reinforced composites by stereolithography with the stress whitening behavior. Mater. Des..

[29] Muller M., Jirku P., Sleger V., Mishra R.K., Hromasova M., Novotny J. (2022). Effect of Infill Density in FDM 3D Printing on Low-Cycle Stress of Bamboo-Filled PLA-Based Material. Polymers.

[30] Yu W.W., Dong L.L., Lei W., Shi J.N. (2020). Rice straw powder/polylactic acid biocomposites for three-dimensional printing. Adv. Compos. Lett..

[31] Zheng L.Y., Liu J.B., Liu R., Xing Y.A., Jiang H. (2021). 3D printing performance of gels from wheat starch, flour and whole meal. Food Chem..

[32] Liu Y.T., Tang T.T., Duan S.Q., Qin Z.Z., Li C., Zhang Z.Q., Liu A.P., Wu D.T., Chen H., Han G.Q. (2020). Effects of sodium alginate and rice variety on the physicochemical characteristics and 3D printing feasibility of rice paste. LWT Food Sci. Technol..

[33] Paggi R.A., Salmoria G.V., Ghizoni G.B., Back H.D., Gindri I.D. (2019). Structure and mechanical properties of 3D-printed cellulose tablets by fused deposition modeling. Int. J. Adv. Manuf. Technol..

[34] Nida S., Moses J.A., Anandharamakrishnan C. (2021). 3D printed food package casings from sugarcane bagasse: A waste valorization study. Biomass Convers. Biorefin..

[35] Latif M., Jiang Y.X., Kumar B., Song J.M., Cho H.C., Kim J. (2022). High Content Nanocellulose 3D-Printed and Esterified Structures with Strong Interfacial Adhesion, High Mechanical Properties, and Shape Fidelity. Adv. Mater. Interfaces.

[36] Shin S., Hyun J. (2021). Rheological properties of cellulose nanofiber hydrogel for high-fidelity 3D printing. Carbohydr. Polym..

[37] Vorobiov V.K., Sokolova M.P., Bobrova N.V., Elokhovsky V.Y., Smirnov M.A. (2022). Rheological properties and 3D-printability of cellulose nanocrystals/deep eutectic solvent electroactive ion gels. Carbohydr. Polym..

[38] Huang H.X., Dean D. (2020). 3-D printed porous cellulose acetate tissue scaffolds for additive manufacturing. Addit. Manuf..

[39] Tuladhar S., Clark S., Habib A. (2023). Tuning Shear Thinning Factors of 3D Bio-Printable Hydrogels Using Short Fiber. Materials.

[40] Murphy C.A., Collins M.N. (2018). Microcrystalline cellulose reinforced polylactic acid biocomposite filaments for 3D printing. Polym. Compos..

[41] Bahcegul E.G., Bahcegul E., Ozkan N. (2020). 3D Printing of Hemicellulosic Biopolymers Extracted from Lignocellulosic Agricultural Wastes. ACS Appl. Polym. Mater..

[42] Xu W.Y., Pranovich A., Uppstu P., Wang X.J., Kronlund D., Hemming J., Oblom H., Moritz N., Preis M., Sandler N. (2018). Novel biorenewable composite of wood polysaccharide and polylactic acid for three dimensional printing. Carbohydr. Polym..

[43] Shi G., Peng X.W., Zeng J.M., Zhong L.X., Sun Y., Yang W., Zhong Y.L., Zhu Y.X., Zou R., Admassie S. (2023). A Liquid Metal Microdroplets Initialized Hemicellulose Composite for 3D Printing Anode Host in Zn-Ion Battery. Adv. Mater..

[44] Dominguez-Robles J., Martin N.K., Fong M.L., Stewart S.A., Irwin N.J., Rial-Hermida M.I., Donnelly R.F., Larraneta E. (2019). Antioxidant PLA Composites Containing Lignin for 3D Printing Applications: A Potential Material for Healthcare Applications. Pharmaceutics.

[45] Ajdary R., Kretzschmar N., Baniasadi H., Trifol J., Seppala J.V., Partanen J., Rojas O.J. (2021). Selective Laser Sintering of Lignin-Based Composites. ACS Sustain. Chem. Eng..

[46] Mimini V., Sykacek E., Syed Hashim S.N.A., Holzweber J., Hettegger H., Fackler K., Potthast A., Mundigler N., Rosenau T. (2019). Compatibility of Kraft Lignin, Organosolv Lignin and Lignosulfonate with PLA in 3D Printing. J. Wood Chem. Technol..

[47] Bonifacio M.A., Cometa S., Cochis A., Scalzone A., Gentile P., Scalia A.C., Rimondini L., Mastrorilli P., De Giglio E. (2022). A bioprintable gellan gum/lignin hydrogel: A smart and sustainable route for cartilage regeneration. Int. J. Biol. Macromol..

[48] Sahai N., Gogoi M., Tewari R.P. (2021). 3D Printed Chitosan Composite Scaffold for Chondrocytes Differentiation. Curr. Med. Imaging..

[49] Pan H.S., Pei F., Ma G.X., Ma N., Zhong L., Zhao L.Y., Hu Q.H. (2022). 3D printing properties of Flammulina velutipes polysaccharide-soy protein complex hydrogels. J. Food Eng..

[50] Feng C.X., Wang Q., Li H., Zhou Q.C., Meng W. (2018). Effects of Pea Protein on the Properties of Potato Starch-Based 3D Printing Materials. Int. J. Food Eng..

[51] Kariz M., Sernek M., Obucina M., Kuzman M.K. (2018). Effect of wood content in FDM filament on properties of 3D printed parts. Mater. Today Commun..

[52] Long H.B., Wu Z.Q., Dong Q.Q., Shen Y.T., Zhou W.Y., Luo Y., Zhang C.Q., Dong X.M. (2019). Mechanical and thermal properties of bamboo fiber reinforced polypropylene/polylactic acid composites for 3D printing. Polym. Eng. Sci..

[53] Yu W.W., Dong L.L., Lei W., Zhou Y.H., Pu Y.Z., Zhang X. (2021). Effects of Rice Straw Powder (RSP) Size and Pretreatment on Properties of FDM 3D-Printed RSP/Poly (lactic acid) Biocomposites. Molecules.

[54] Ambone T., Torris A., Shanmuganathan K. (2020). Enhancing the mechanical properties of 3D printed polylactic acid using nanocellulose. Polym. Eng. Sci..

[55] Ryu J.A., Lee J.M., Eom T.J. (2019). Comparison of 3D Printer Application and Strength Property Using Polylactic Acid Filaments with Lignin-free and -rich MFC. Palpu Chongi Gisul J. Korea Tech. Assoc. Pulp Pap. Ind..

[56] Yang T.C., Yeh C.H. (2020). Morphology and Mechanical Properties of 3D Printed Wood Fiber/Polylactic Acid Composite Parts Using Fused Deposition Modeling (FDM): The Effects of Printing Speed. Polymers.

[57] Zhang Y.H., Fang J., Li J., Guo Y.L., Wang Q.W. (2017). The Effect of Carbon Nanotubes on the Mechanical Properties of Wood Plastic Composites by Selective Laser Sintering. Polymers.

[58] Ajdary R., Huan S.Q., Ezazi N.Z., Xiang W.C., Grande R., Santos H.A., Rojas O.J. (2019). Acetylated Nanocellulose for Single-Component Bioinks and Cell Proliferation on 3D-Printed Scaffolds. Biomacromolecules.

[59] Espinosa E., Filgueira D., Rodriguez A., Chinga-Carrasco G. (2019). Nanocellulose-Based Inks-Effect of Alginate Content on the Water Absorption of 3D Printed Constructs. Bioengineering.

[60] Rosa R.P., Rosace G., Arrigo R., Malucelli G. (2023). Preparation and characterization of a fully biobased resin system for 3d-printing, suitable for replacing fossil-based acrylates. J. Polym. Res..

[61] Travitzky N., Windsheimer H., Fey T., Greil P. (2008). Preceramic Paper-Derived Ceramics. J. Am. Ceram. Soc..

[62] Liu Q.W., Yang C.M., Miao Q., Liu Y.F., Liu J.Q., Yu W.J. (2020). Experimental Study on Inert Gas-assisted Laser Cut Veneer Based on LOM. J. Renew. Mater..

[63] Bouzidi K., Chaussy D., Gandini A., Bongiovanni R., Beneventi D. (2022). 3D printable fully biomass-based composite using poly (furfuryl alcohol) as binder and cellulose as a filler. Carbohyd. Polym..

[64] Rosenthal M., Henneberger C., Gutkes A., Bues C.T. (2017). Liquid Deposition Modeling: A promising approach for 3D printing of wood. Eur. J. Wood Prod..

[65] Liu L.X., Lin M.H., Xu Z., Lin M.Q. (2019). Polylactic Acid-based Wood-plastic 3D Printing Composite and its Properties. BioResources.

[66] Rahim T.N.A.T., Abdullah A.M., Akil H.M. (2019). Recent Developments in Fused Deposition Modeling-Based 3D Printing of Polymers and Their Composites. Polym. Rev..

[67] Tascioglu E., Kitay O., Keskin A.O., Kaynak Y. (2022). Effect of printing parameters and post-process on surface roughness and dimensional deviation of PLA parts fabricated by extrusion-based 3D printing. J. Braz. Soc. Mech. Sci. Eng..

[68] Fico D., Rizzo D., Casciaro R., Corcione C.E. (2022). A Review of Polymer-Based Materials for Fused Filament Fabrication (FFF): Focus on Sustainability and Recycled Materials. Polymers.

[69] Cano-Vicent A., Tambuwala M.M., Hassan S.S., Barh D., Aljabali A.A.A., Birkett M., Arjunan A., Serrano-Aroca A. (2021). Fused deposition modelling: Current status, methodology, applications and future prospects. Addit. Manuf..

[70] Baechle-Clayton M., Loos E., Taheri M., Taheri H. (2022). Failures and Flaws in Fused Deposition Modeling (FDM) Additively Manufactured Polymers and Composites. J. Compos. Sci..

[71] Markstedt K., Sundberg J., Gatenholm P. (2014). 3D Bioprinting of Cellulose Structures from an Ionic Liquid. 3D Print. Addit. Manuf..

[72] Hu X.Z., Yang Z.J., Kang S.X., Jiang M., Zhou Z.W., Gou J.H., Hui D., He J. (2020). Cellulose hydrogel skeleton by extrusion 3D printing of solution. Nanotechnol. Rev..

[73] Wang J.P., Chiappone A., Roppolo I., Shao F., Fantino E., Lorusso M., Rentsch D., Dietliker K., Pirri C.F., Grutzmacher H. (2018). All-in-One Cellulose Nanocrystals for 3D Printing of Nanocomposite Hydrogels. Angew. Chem. Int. Edit..

[74] Hossain S.S., Lu K.T.Y. (2023). Recent progress of alumina ceramics by direct ink writing: Ink design, printing and post-processing. Ceram. Int..

[75] Saadi M.A.S.R., Maguire A., Pottackal N.T., Thakur M.S.H., Ikram M.M., Hart A.J., Ajayan P.M., Rahman M.M. (2022). Direct Ink Writing: A 3D Printing Technology for Diverse Materials. Adv. Mater..

[76] Hou Z.Z., Lu H., Yang L.X., Gao Y. (2021). Direct Ink Writing of Materials for Electronics-Related Applications: A Mini Review. Front. Mater..

[77] Sutton J.T., Rajan K., Harper D.P., Chmelu S.C. (2018). Lignin-Containing Photoactive Resins for 3D Printing by Stereolithography. ACS Appl. Mater. Interfaces.

[78] Quan H.Y., Zhang T., Xu H., Luo S., Nie J., Zhu X.Q. (2020). Photo-curing 3D printing technique and its challenges. Bioact. Mater..

[79] Rosa R.P., Rosace G. (2021). Nanomaterials for 3D Printing of Polymers via Stereolithography: Concept, Technologies, and Applications. Macromol. Mater. Eng..

[80] Han W., Kong L.B., Xu M. (2022). Advances in selective laser sintering of polymers. Int. J. Extreme Manuf..

[81] Chin S.Y., Dikshit V., Priyadarshini B.M., Zhang Y. (2020). Powder-Based 3D Printing for the Fabrication of Device with Micro and Mesoscale Features. Micromachines.

[82] Awad A., Fina F., Goyanes A., Gaisford S., Basit A.W. (2020). 3D printing: Principles and pharmaceutical applications of selective laser sintering. Int. J. Pharm..

[83] Lupone F., Padovano E., Casamento F., Badini C. (2022). Process Phenomena and Material Properties in Selective Laser Sintering of Polymers: A Review. Materials.

[84] Pilipovic A., Raos P., Sercer M. (2011). Experimental testing of quality of polymer parts produced by laminated object manufacturing—LOM. Teh. Vjesn..

[85] Wang M.R., Jin G.R., He W.D., Nan F.Q. (2022). 3D printing of gun propellants based on laminated object manufacturing. Mater. Manuf. Process..

[86] Dizon J.R.C., Gache C.C.L., Cascolan H.M.S., Cancino L.T., Advincula R.C. (2021). Post-Processing of 3D-Printed Polymers. Technologies.

[87] Postiglione G., Natale G., Griffini G., Levi M., Turri S. (2015). Conductive 3D microstructures by direct 3D printing of polymer/carbon nanotube nanocomposites via liquid deposition modeling. Compos. Part A Appl. Sci. Manuf..

[88] Bi H.J., Jia X., Ye G.Y., Ren Z.C., Yang H.Y., Guo R., Xu M., Cai L.P., Huang Z.H. (2020). Three-Dimensional-Printed Shape Memory Biomass Composites for Thermal-Responsive Devices. 3D Print Addit. Manuf..

[89] Tanase-Opedal M., Espinosa E., Rodriguez A., Chinga-Carrasco G., Rodríguez A. (2019). Lignin: A Biopolymer from Forestry Biomass for Biocomposites and 3D Printing. Materials.

[90] Le Guen M.J., Hill S., Smith D., Theobald B., Gaugler E., Barakat A., Mayer-Laigle C. (2019). Influence of Rice Husk and Wood Biomass Properties on the Manufacture of Filaments for Fused Deposition Modeling. Front. Chem..

[91] Mohan D., Bakir A.N., Sajab M.S., Bakarudin S.B., Mansor N.N., Roslan R., Kaco H. (2021). Homogeneous distribution of lignin/graphene fillers with enhanced interlayer adhesion for 3D printing filament. Polym. Compos..

[92] Tao Y.B., Wang H.L., Li Z.L., Li P., Shi S.Q. (2017). Development and Application of Wood Flour-Filled Polylactic Acid Composite Filament for 3D Printing. Materials.

[93] Bhagia S., Lowden R.R., Erdman D., Rodriguez M., Haga B.A., Solano I.R.M., Gallego N.C., Pu Y.Q., Muchero W. (2020). Tensile properties of 3D-printed wood-filled PLA materials using poplar trees. Appl. Mater. Today.

[94] Rahmatabadi D., Aberoumand M., Soltanmohammadi K., Soleyman E., Ghasemi I., Baniassadi M., Abrinia K., Bodaghi M., Baghani M. (2023). Toughening PVC with Biocompatible PCL Softeners for Supreme Mechanical Properties, Morphology, Shape Memory Effects, and FFF Printability. Macromol. Mater. Eng..

[95] Yu W.W., Shi J.N., Sun L.W., Lei W. (2022). Effects of Printing Parameters on Properties of FDM 3D Printed Residue of Astragalus/Polylactic Acid Biomass Composites. Molecules.

[96] Wang Q.Q., Ji C.C., Sun L.S., Liu J. (2020). Cellulose nanofibrils filled poly (Lactic Acid) biocomposite filament for FDM 3D printing. Molecules.

[97] Dezaki M.L., Ariffin M.K.A.M., Serjouei A., Zolfagharian A., Hatami S., Bodaghi M. (2021). Influence of Infill Patterns Generated by CAD and FDM 3D Printer on Surface Roughness and Tensile Strength Properties. Appl. Sci..

[98] Thibaut C., Denneulin A., du Roscoat S.R., Beneventi D., Orgeas L., Chaussy D. (2019). A fibrous cellulose paste formulation to manufacture structural parts using 3D printing by extrusion. Carbohydr. Polym..

[99] Håkansson K.M.O., Henriksson I.C., Vazquez C.D., Kuzmenko V., Markstedt K., Enoksson P., Gatenholm P. (2016). Solidification of 3D Printed Nanofibril Hydrogels into Functional 3D Cellulose Structures. Adv. Mater. Technol..

[100] Li V.C.F., Dunn C.K., Zhang Z., Deng Y.L., Qi H.J. (2017). Direct Ink Write (DIW) 3D Printed Cellulose Nanocrystal Aerogel Structures. Sci. Rep..

[101] Lai C.W., Yu S.S. (2020). 3D Printable Strain Sensors from Deep Eutectic Solvents and Cellulose Nanocrystals. ACS Appl. Mater. Interfaces.

[102] Jiang B., Yao Y.G., Liang Z.Q., Gao J.L., Chen G.C., Xia Q.Q., Mi R.Y., Jiao M.L., Wang X.Z., Hu L.B. (2020). Lignin-Based Direct Ink Printed Structural Scaffolds. Small.

[103] Lebedevaite M., Ostrauskaite J., Skliutas E., Malinauskas M. (2019). Photoinitiator Free Resins Composed of Plant-Derived Monomers for the Optical µ-3D Printing of Thermosets. Polymers.

[104] Barkane A., Platnieks O., Jurinovs M., Kasetaite S., Ostrauskaite J., Gaidukovs S., Habibi Y. (2021). UV-Light Curing of 3D Printing Inks from Vegetable Oils for Stereolithography. Polymers.

[105] Wu Y.C., Advincula P.A., Giraldo-Londono O., Yu Y.K., Xie Y.C., Chen Z.R., Huang G.l., Tour J.M., Lin J. (2022). Sustainable 3D Printing of Recyclable Biocomposite Empowered by Flash Graphene. ACS Nano.

[106] Romero-Ocana I., Molina S.I. (2022). Cork photocurable resin composite for Stereolithography (SLA): Influence of cork particle size on mechanical and thermal properties. Addit. Manuf..

[107] Rosace G., Rosa R.P., Arrigo R., Malucelli G. (2021). Photosensitive acrylates containing bio-based epoxy-acrylate soybean oil for 3D printing application. J. Appl. Polym. Sci..

[108] Guit J., Tavares M.B.L., Hul J., Ye C.N., Loos K., Jager J., Folkersma R., Voet V.S.D. (2020). Photopolymer Resins with Biobased Methacrylates Based on Soybean Oil for Stereolithography. ACS Appl. Polym. Mater..

[109] Sutton J.T., Rajan k., Harper D.P., Chmely S.C. (2021). Improving UV Curing in Organosolv Lignin-Containing Photopolymers for Stereolithography by Reduction and Acylation. Polymers.

[110] Shirazi S.F.S., Gharehkhani S., Mehrali M., Yarmand H., Metselaar H.S.C., Kadri N.A., Osman N.A.A. (2015). A review on powder-based additive manufacturing for tissue engineering: Selective laser sintering and inkjet 3D printing. Sci. Technol. Adv. Mater..

[111] Zeng W.L., Guo Y.L., Liu Y., Gong Y.P., Zhao P.F., Li C.L., Zhao J.W. (2013). A New Rice Husk-Plastic Composite Powder Preparation Process for Selective Laser Sintering Fabrication. Adv. Mater. Res..

[112] Guo S., Li J., Zhang L., Li Y.C. (2023). Preparation of high-porosity biomass-based carbon electrodes by selective laser sintering. Mater. Lett..

[113] Zeng W.L., Guo Y.L., Jiang K.Y., Yu Z.X., Liu Y., Shen Y.D., Deng J.R., Wang P.X. (2013). Laser intensity effect on mechanical properties of wood-plastic composite parts fabricated by selective laser sintering. J. Thermoplast. Compos. Mater..

[114] Zhang Y.H., Cui Y.H., Wang S., Zhao X.W., Wang F.M., Wu G.H. (2020). Effect of microwave treatment on bending properties of carbon nanotube/wood plastic composites by selective laser sintering. Mater. Lett..

[115] Zhang Y.H., Wang F.M., Zhang Y.F., Li J., Guo Y.L. (2021). Effect of Al powder on mechanical properties and microstructure of wood-plastic composites by selective laser sintering. Mater. Today Commun..

[116] Sonmez F.O., Hahn H.T. (1998). Thermomechanical analysis of the laminated object manufacturing (LOM) process. Rapid Prototyp. J..

[117] Tao Y.B., Yin Q., Li P. (2020). An Additive Manufacturing Method Using Large-Scale Wood Inspired by Laminated Object Manufacturing and Plywood Technology. Polymers.

[118] Kariz M., Sernek M., Kuzman M.K. (2016). Use of wood powder and adhesive as a mixture for 3D printing. Eur. J. Wood Wood Prod..

[119] Pitt K., Lopez-Botello O., Lafferty A.D., Todd I., Mumtaz K. (2017). Investigation into the material properties of wooden composite structures with in-situ fibre reinforcement using additive manufacturing. Compos. Sci. Technol..

[120] Arif Z.U., Khalid M.Y., Zolfagharian A., Bodaghi M. (2022). 4D bioprinting of smart polymers for biomedical applications: Recent progress, challenges, and future perspectives. React. Funct. Polym..

[121] Jo G.H., Lim W.S., Kim H.W., Park H.J. (2021). Post-processing and printability evaluation of red ginseng snacks for three-dimensional (3D) printing. Food Biosci..

[122] Tetik H., Zhao K.R., Shah N.A., Lin D. (2021). 3D freeze-printed cellulose-based aerogels: Obtaining truly 3D shapes, and functionalization with cross-linking and conductive additives. J. Manuf. Process..

[123] Garcia E.A., Ayranci C., Qureshi A.J. (2020). Material Property-Manufacturing Process Optimization for Form 2 Vat-Photo Polymerization 3D printers. J. Manuf. Mater. Process..

[124] Liu Z.S., Knetzer D.A., Wang J.F., Chu F.X., Lu C.W., Calvert P.D. (2021). 3D printing acrylated epoxidized soybean oil reinforced with functionalized cellulose by UV curing. J. Appl. Polym. Sci..

[125] Zhao Z.C., Wu H., Liu X.D., Kang D.S., Xiao Z.H., Lin Q.Q., Zhang A.H. (2022). Synthesis and characterization of tung oil-based UV curable for three-dimensional printing resins. RSC Adv..

[126] Chen K.J., Kuang X., Li V., Kang G.Z., Qi H.J. (2018). Fabrication of tough epoxy with shape memory effects by UV-assisted direct-ink write printing. Soft Matter.

[127] Pascual-Gonzalez C., San Martin P., Lizarralde I., Fernandez A., Leon A., Lopes C.S., Fernandez-Biazquez J.P. (2021). Post-processing effect on microstructure, interlaminar and thermal properties of 3D printed continuous carbon fiber composites. Compos. Part B Eng..

[128] Yang Z.Z., Wu G.M., Wang S.Q., Xu M., Feng X.H. (2018). Dynamic Postpolymerization of 3D-Printed Photopolymer Nanocomposites: Effect of Cellulose Nanocrystal and Postcure Temperature. J. Polym. Sci. Part B Polym. Phys..

[129] Ge Y., Zhang T., Zhou B., Wang H.Q., Zhang Z.H., Shen J., Du A. (2018). Nanostructured resorcinol-formaldehyde ink for 3D direct writing. J. Mater. Res..

[130] Armstrong C. Post Processing for FDM Printed Parts. https://www.hubs.com/knowledge-base/post-processing-FFF-printed-parts/#weld.

[131] Sun Z., Lu Y.H., Zhao Q., Wu J.J. (2022). A new stereolithographic 3D printing strategy for hydrogels with a large mechanical tunability and self-weldability. Addit. Manuf..

[132] Yang E., Miao S.D., Zhong J., Zhang J.Y., Mills D.K., Zhang L.G. (2018). Bio-Based Polymers for 3D Printing of Bioscaffolds. Polym. Rev..

[133] Athukoralalage S.S., Balu R., Dutta N.K., Choudhury N.R. (2019). 3D Bioprinted Nanocellulose-Based Hydrogels for Tissue Engineering Applications: A Brief Review. Polymers.

[134] Kanikireddy V., Varaprasad K., Jayaramudu T., Karthikeyan C., Sadiku R. (2020). Carboxymethyl cellulose-based materials for infection control and wound healing: A review. Int. J. Biol. Macromol..

[135] Surini S., Bimawanti Y., Kurniawan A. (2022). The Application of Polymers in Fabricating 3D Printing Tablets by Fused Deposition Modeling (FDM) and The Impact on Drug Release Profile. Pharm. Sci..

[136] Zamboulis A., Michailidou G., Koumentakou I., Bikiaris D.N. (2022). Polysaccharide 3D Printing for Drug Delivery Applications. Pharmaceutics.

[137] Miao S.D., Zhu W., Castro N.J., Nowicki M., Zhou X., Cui H.T., Fisher J.P., Zhang L.G. (2016). 4D printing smart biomedical scaffolds with novel soybean oil epoxidized acrylate. Sci. Rep..

[138] Calì M., Pascoletti G., Gaeta M., Milazzo G., Ambu R. (2020). New filaments with natural fillers for FDM 3D printing and their applications in biomedical field. Procedia Manuf..

[139] Khaled S.A., Burley J.C., Alexander M.R., Yang J., Roberts C.J. (2015). 3D printing of five-in-one dose combination polypill with defined immediate and sustained release profiles. J. Control. Release.

[140] Rees A., Powell L.C., Chinga-Carrasco G., Gethin D.T., Syverud K., Hill K.E., Thomas D.W. (2015). 3D Bioprinting of Carboxymethylated-Periodate Oxidized Nanocellulose Constructs for Wound Dressing Applications. Biomed Res. Int..

[141] Leppiniemi J., Lahtinen P., Paajanen A., Mahlberg R., Metsa-Kortelainen S., Pinornaa T., Pajari H., Vikholm-Lundin I., Pursula P., Hytonen V.P. (2017). 3D-printable bioactivated nanocellulose-alginate hydrogels. Acs Appl. Mater. Interfaces.

[142] Xu W.Y., Zhang X., Yang P.R., Langvik O., Wang X.J., Zhang Y.C., Cheng F., Osterberg M., Willfor S., Xu C.L. (2019). Surface Engineered Biomimetic Inks Based on UV Cross-Linkable Wood Biopolymers for 3D Printing. ACS Appl. Mater. Interfaces.

[143] Schwarz S., Avila H.M., Rotter N., Gatenholm P. (2016). 3D Bioprinting of Human Chondrocyte-laden Nanocellulose Hydrogels for Patient-specific Auricular Cartilage Regeneration. Tissue Eng. Part A.

[144] Jessop Z.M., Al-Sabah A., Gao N., Kyle S., Thomas B., Badiei N., Hawkins K., Whitaker I.S. (2019). Printability of pulp derived crystal, fibril and blend nanocellulose-alginate bioinks for extrusion 3D bioprinting. Biofabrication.

[145] Zhang T.Y., Li X., Asher E., Deng S.X., Sun X.L., Yang J. (2018). Paper with Power: Engraving 2D Materials on 3D Structures for Printed, High-Performance, Binder-Free, and All-Solid-State Supercapacitors. Adv. Funct. Mater..

[146] Zhu Y.X., Qin J.D., Shi G., Sun C., Ingram M., Qian S.S., Lu J., Zhang S.Q., Zhong Y.L. (2022). A focus review on 3D printing of wearable energy storage devices. Carbon Energy.

[147] Hamad W.Y. (2016). Photonic and Semiconductor Materials Based on Cellulose Nanocrystals. Adv. Polym. Sci..

[148] Ee L.Y., Li S.F.Y. (2021). Recent advances in 3D printing nanocellulose: Structure, preparation and application prospects. Nanoscale Adv..

[149] Cao D.X., Xing Y.J., Tantratian K., Wang X., Ma Y., Mukhopadhyay A., Cheng Z., Zhang Q., Jiao Y.C., Chen L. (2019). 3D Printed High-Performance Lithium Metal Microbatteries Enabled by Nanocellulose. Adv. Mater..

[150] Wang C.S., Wu S.Q., Li Z.Q., Chen S., Chen A.N., Yan C.Z., Shi Y.S., Zhang H.B., Fan P.Y. (2022). 3D printed porous biomassderived SiCnw/SiC composite for structurefunction integrated electromagnetic absorption. Virtual Phys. Prototyp..

[151] Hsieh M.C., Kim C., Nogi M., Suganuma K. (2013). Electrically conductive lines on cellulose nanopaper for flexible electrical devices. Nanoscale.

[152] Huang S.Q., Su S.Y., Gan H.B., Wu L.J., Lin C.H., Xu D.Y., Zhou H.F., Lin X.L., Qin Y.L. (2019). Facile fabrication and characterization of highly stretchable lignin-based hydroxyethyl cellulose self-healing hydrogel. Carbohydr. Polym..

[153] Qian J., Chen Q.Y., Hong M., Xie W.Q., Jing S.S., Bao Y.H., Chen G., Pang Z.Q., Hu L.B., Li T. (2022). Toward stretchable batteries: 3D-printed deformable electrodes and separator enabled by nanocellulose. Mater. Today.

[154] Saha M.C., Maity K., Mondal A. (2023). Cellulose Nanocrystal-Based All-3D-Printed Pyro-Piezoelectric Nanogenerator for Hybrid Energy Harvesting and Self-Powered Cardiorespiratory Monitoring toward the Human-Machine Interface. Acs Appl. Mater. Interfaces.

[155] Henke K., Treml S. (2013). Wood based bulk material in 3D printing processes for applications in construction. Eur. J. Wood Wood Prod..

[156] Yoshida H., Igarashi T., Obuchi Y., Takami Y., Sato J., Araki M., Miki M., Nagata K., Sakai K., Igarashi S. (2015). Architecture-Scale Human-Assisted Additive Manufacturing. ACM Trans. Grap..

[157] Zhao X.H., Tekinalp H., Meng X.Z., Ker D., Benson B., Pu Y.Q., Ragauskas A.J., Wang Y., Li K., Webb E. (2019). Poplar as Biofiber Reinforcement in Composites for Large-Scale 3D Printing. ACS Appl. Bio Mater..

[158] Alqenaee A., Memari A. (2022). Experimental study of 3D printable cob mixtures. Constr. Build. Mater..

[159] Yang S.G., Du P. (2022). The application of 3D printing technology in furniture design. Sci. Program..

[160] Bhagia S., Bornani K., Agrawal R., Satlewal A., Durkovic J., Lagana R., Bhagia M., Yoo C.G., Zhao X.H., Kunc V. (2021). Critical review of FDM 3D printing of PLA biocomposites filled with biomass resources, characterization, biodegradability, upcycling and opportunities for biorefineries. Appl. Mater. Today.

[161] Feng X.H., Wu Z.H., Sang R.J., Wang F., Zhu Y.Y., Wu M.J. (2019). Surface design of wood-based board to imitate wood texture using 3D printing technology. BioResources.

[162] Sang R.J., Manley A.J., Wu Z.H., Feng X.H. (2020). Digital 3D Wood Texture: UV-Curable Inkjet Printing on Board Surface. Coatings.

[163] Lille M., Nurmela A., Nordlund E., Metsa-Kortelainen S., Sozer N. (2018). Applicability of protein and fiber-rich food materials in extrusion-based 3D printing. J. Food Eng..

[164] Shahbazi M., Jager H., Ettelaie R. (2022). A Promising Therapeutic Soy-Based Pickering Emulsion Gel Stabilized by a Multifunctional Microcrystalline Cellulose: Application in 3D Food Printing. J. Agric. Food Chem..

[165] Letras P., Oliveira S., Varela J., Nunes M.C., Raymundo A. (2022). 3D printed gluten-free cereal snack with incorporation of Spirulina (*Arthrospira platensis*) and/or Chlorella vulgaris. Algal Res..

